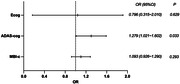# 500nm blue‐green light therapy for cognitive function in Alzheimer’s continuum and factors affecting efficacy

**DOI:** 10.1002/alz.090108

**Published:** 2025-01-09

**Authors:** Ziqi Wang, Junlan Yang, Qiansen Feng, Linlin Li, Lei Chen, Jiayu Wang, Jiajing Wu

**Affiliations:** ^1^ The Clinical Hospital of Chengdu Brain Science Institute, MOE Key Lab for Neuroinformation, School of Life Science and Technology, University of Electronic Science and Technology of China, Chengdu China; ^2^ The Fourth People’s Hospital of Chengdu, Chengdu China; ^3^ The Fourth People’s Hospital of Pengzhou, Chengdu China; ^4^ Nursing School of Zunyi Medical University, Zunyi China

## Abstract

**Background:**

Light therapy has emerged as an effective method for Alzheimer’s disease (AD). However, the efficacy on cognitive function in Alzheimer’s continuum and factors affecting efficacy have not been established. The study aimed to evaluate the efficacy of 500 nm blue‐green light therapy on cognitive function for Alzheimer’s continuum, and the main factors affecting light therapy efficacy were analyzed.

**Method:**

Forty‐two participants were enrolled in this studies, including 13 healthy control [HC, equal to 0 standard deviation, (SD)], 9 subtle cognitive impairment (SCI, ≥0.5 SD), 8 early mild cognitive impairment (eMCI, ≥1.0 SD), 6 late MCI (lMCI, ≥1.5 SD), 6 dementia of AD (dAD, ≥2.0 SD). All the participants treated with 500 nm blue‐green light for 50 minutes with 4 weeks in the morning. A reduction of 4 or more points in the Alzheimer’s Disease Assessment Scale‐cognitive subscale (ADAS‐cog) scores was considered valid for cognitive improvement.

**Result:**

The total efficacy after light therapy was 16.7%. The efficacy in HC, SCI, eMCI, lMCI and dAD after light therapy were 15.4%, 11.1%, 12.5%, 16.7% and 33.3%, respectively. Light therapy efficacy was not significantly correlated with the participant’s sex, age, diagnostic type, education, apolipoprotein E epsilon 4, the Mini‐mental State Examination scores; the Montreal Cognitive Assessment‐Basic scores, Hamilton Anxiety Scale scores, Hamilton Depression Scale scores, or the Pittsburgh Sleep Quotient Index. In multivariate analysis, the ADAS‐cog scores before light therapy (OR 1.279, 95% CI 1.021∼1.602, p = 0.033) was an independent predictor, while the Everyday Cognition (ECog) scores and the Mild Behavioral Impairment Checklist (MBI‐C) scores before light therapy didn’t influence the efficacy.

**Conclusion:**

Four weeks of 500 nm blue‐green light therapy had different efficacy on cognitive function for Alzheimer’s continuum. Baseline cognitive status may influence light therapy efficacy.